# Parotid gland shrinkage during IMRT predicts the time to Xerostomia resolution

**DOI:** 10.1186/s13014-015-0331-x

**Published:** 2015-01-17

**Authors:** Giuseppe Sanguineti, Francesco Ricchetti, Binbin Wu, Todd McNutt, Claudio Fiorino

**Affiliations:** Department of Radiation Oncology, Johns Hopkins University, Baltimore, MD USA; Department of Molecular Radiation Sciences, Johns Hopkins University, Baltimore, MD USA; Department of Medical Physics, Ospedale San Raffaele, Milan, Italy

**Keywords:** Parotid gland, IMRT, Shrinkage

## Abstract

**Purpose:**

To assess the impact of mid-treatment parotid gland shrinkage on long term xerostomia during IMRT for oropharyngeal SCC.

**Methods and materials:**

All patients treated with IMRT at a single Institution from November 2007 to June 2010 and undergoing weekly CT scans were selected. Parotid glands were contoured retrospectively on the mid treatment CT scan. For each parotid gland, the percent change relative to the planning volume was calculated and combined as weighted average. Patients were considered to be xerostomic if developed GR2+ dry mouth according to CTCAE v3.0. Predictors of the time to xerostomia resolution or downgrade to 1 were investigated at both uni- and multivariate analysis.

**Results:**

85 patients were selected. With a median follow up of 35.8 months (range: 2.4-62.6 months), the actuarial rate of xerostomia is 26.2% (SD: 5.3%) and 15.9% (SD: 5.3%) at 2 and 3 yrs, respectively. At multivariate analysis, mid-treatment shrink along with weighted average mean parotid dose at planning and body mass index are independent predictors of the time to xerostomia resolution. Patients were pooled in 4 groups based on median values of both mid-treatment shrink (cut-off: 19.6%) and mean WA parotid pl-D (cut-off: 35.7 Gy). Patients with a higher than median parotid dose at planning and who showed poor shrinkage at mid treatment are the ones with the outcome significantly worse (3-yr rate of xerostomia ≈ 50%) than the other three subgroups (3-yr rate of xerostomia ≈ 10%).

**Conclusion:**

For a given planned dose, patients whose parotids significantly shrink during IMRT are less likely to be long-term supplemental fluids dependent.

## Introduction

Long-term dry mouth is a debilitating side effect of radiotherapy for head and neck cancer. More than a decade ago, Eisbruch et al. explored for the first time the existence of a direct relationship between the dose received by parotids and salivary output after radiotherapy [1]. After this early work, many studies investigated this issue, leading to a quite good consensus around the parallel behavior of parotids and the value of the mean dose to predict xerostomia [2]; consequently, this knowledge led to limit prospectively the dose to the parotid glands throughout IMRT. Data from randomized studies have confirmed the clinical validity of this approach [3]. However, despite ‘dosimetric’ sparing of the parotids, 20-25% of patients still develop long-term xerostomia. Whether this reflects a different intrinsic radiosensitivity among patients, the added damage to other major/minor glands or the inability to correctly predict for the real dose delivered to the parotids is unclear.

We and others have shown that the parotid glands undergo, on average, a ≈ 30% percent reduction in volume during radiotherapy [4,5] and this, along with their migration medially towards the high dose region [6], may actually result in a higher than planned cumulative dose [7]. On the other hand, early parotid gland shrinkage during treatment has been found to be independently correlated to the dose at planning [5] and thus volumetric reduction may just indirectly reflect a higher dose at planning. Regardless the mechanism, we recently found a correlation between volumetric parotid variations during IMRT and acute xerostomia in 24 patients [8], suggesting that morphologic changes may have detectable clinical relevance. While these data need confirmation in a larger cohort, they also raise the possibility that early volumetric changes may be used to predict for late or long term toxicity.

In the present study we investigated the impact, if any, of parotid gland shrinkage at mid-treatment on long term xerostomia after accounting for the parotid dose at planning and other potential confounders.

## Materials and methods

### Patients and treatment

For the purpose of the present study, that has been approved by the local IRB, patients treated with definitive IMRT ± chemotherapy for oropharyngeal SCC and who underwent weekly (KV)CT scans in addition to the planning CT (pl-CT) as part of an internal QA program at Johns Hopkins University from November 2007 to June 2010 were selected [5].

All patients underwent 3 dose level painting IMRT with 68.25/70 Gy prescribed to macroscopic disease (CTV1), 63 Gy to microscopic high risk disease (CTV2) and 58.1 Gy to microscopic low risk disease (CTV3). All doses were given in 35 fractions over 7 weeks. Each CTV was expanded by 5 mm to the corresponding PTV. IMRT was administered with a 9-field step and shoot technique. Thirty-five patients (41.2%) underwent daily CBCTs (IGRT). Dose volume objectives were placed on both primary (brain, brainstem, cord + 4 mm) and secondary (mandible, parotids and larynx) organs at risk. For the parotids, the dose volume objective was set at V30 Gy < 50%. All patients were treated comprehensively on both sides of the neck. Parotid glands were retrospectively contoured on the mid treatment CT scan under the supervision of the same physician (G.S.) and blindly to clinical outcome. Patients with parotid glands grossly infiltrated by tumor were excluded. Major salivary glands were arbitrarily labeled as H or L whether they were planned to receive the higher (H) or lower (L) mean dose within a given patient.

For each parotid gland, the percent change in volume relative to the planning volume was calculated at the CT closest to mid-treatment. Each side was entered separately or as a combined weighted (by the volume at planning) average. Regarding the mean dose at planning of major salivary glands, each organ was entered separately or as combined mean dose. The latter was computed as the weighted (by the volume at planning) average of the mean doses to each gland.

### Statistics

According to internal guidelines, patients were scheduled to be seen also in the department of Radiation Oncology at regular follow up intervals (3, 6, 12, 18, 24, 36, 48 and 60 months after treatment completion).

As previously reported [9], at each follow up examination, dry mouth/xerostomia was scored and digitally recorded by one observer (G.S.) according to Common Terminology Criteria for Adverse Events v 3.0. Grade 1 (GR1) would include patients with dry or thick saliva without significant dietary alteration; Grade 2 (GR2), symptomatic patients with significant oral intake alteration (i.e. copious water or other lubricants). Particular attention was placed to whether or not the patient was carrying supplemental fluids at the time of the office examination. Salivary flow was not measured routinely. The main purpose of the present study is to investigate the predictive role of parotid gland shrinkage at mid-treatment along with selected patient-, tumor- and treatment-related characteristics on the time to xerostomia resolution (GR0) or downgrade from GR2+ to GR1. This was computed from the end of radiotherapy to the date of the first visit in which objective and subjective assessment parameters were consistent with GR0-1 dry mouth or the last follow up for censored observations (patients without xerostomia resolution). Observations obtained after locoregional failure were disregarded, with the exception of residual disease at complementary neck dissection. For patients who did not develop GR2+ xerostomia, the time to xerostomia resolution/downgrade was set at zero. In patients with bouncing scores at subsequent follow ups (i.e. GR2 → GR1 → GR2), only the worst/latest score was considered, disregarding earlier improvements.

Actuarial rates were computed with the Kaplan Meier method and compared with the log rank test. Covariates with a p-value lower than 0.2 at univariate analysis were entered into a Cox proportional hazard ratio at multivariate analysis. A backward selection procedure based on the likelihood ratio test was used to select variables. Patients were pooled in 4 groups based on median values of both mid-treatment shrink and mean weighted average parotid planning dose. All tests were two-sided and statistical significance was claimed for a p value < 0.05. All analyses were performed using GraphPad (version 5.0, GraphPad Software Inc, San Diego, CA) and SPSS (version 17.0, SPSS Inc, Chicago, IL).

## Results

The analyzed patient population consists of 85 patients. Main patient, tumor and treatment characteristics have been reported previously and are summarized in Table 1 [5]. Briefly, median age was 57.5 years (range: 30–81 yrs) while median body mass index (BMI) was 28.45 kg/m^2^ (range: 20.45-41.82 kg/m^2^). Eighty-two of 85 patients completed IMRT as prescribed with 3 patients missing 1–2 fractions (total dose: 66–68 Gy). Median overall treatment time was 7.0 weeks (range: 6.6-8.7 wks). The median relative weight change during treatment was −8.9% (range: +5.6% to −24.7%).

**Table 1:** **Selected patient, tumor and treatment characteristics**

**Characteristic**	**Stratification**	**# pts**	**%**
Sex	Male	77	90.6
	Female	8	9.4
Primary tumor site	Tonsil	37	43.5
	Base of tongue	46	54.1
	Pharyngeal wall	2	2.4
AJCC stage	I	2	2.4
	II	7	8.2
	III	8	9.4
	IV	68	80.0
Smoking at diagnosis	No	67	78.8
	Yes	18	21.2
Smoking during follow up	No	73	85.9
	Yes	12	14.1
PEG	No	17	20.0
	Yes	68	80.0
Px dose to PTV1	68.25 Gy	5	5.9
	70 Gy	80	94.1
Chemotherapy	Concomitant (Platin-based)	63	74.1
	Induction + Concomitant	7	8.2
	Cetuximab	5	5.9
	None	10	11.8

Patients underwent the pre-treatment planning CT at a median time of 19 days (range: 10–25 days) before treatment initiation; the median number of fractions from treatment start to mid-treatment scan were 16 (range: 13–21 fxs) [5].

Dosimetric parotid gland data at planning are summarized in Table 2. Mean submandibular doses at planning were as follows: median contralateral SMG mean dose (85 pts), 59.3 Gy (range: 12.8/71.4 Gy); median ipsilateral SMG mean dose (80 pts), 68.4 Gy (range: 50.0/73.1 Gy); median weighted average SMG mean dose (80 pts), 63.6 Gy (range: 35.4/71.6 Gy). Median weighted average combined parotid and submandibular mean dose at planning was 42.4 Gy (range: 22.7/58.7 Gy). The latter was closely correlated to the WA parotid (Spearman rho = 0.88, p < 0.001) rather than the WA SMG (rho = 0.17, p = 0.13) mean dose. Median oral mucosa mean dose at planning was 53.1 Gy (range: 35.7/69 Gy).

**Table 2:** **Dosimetric parotid gland data at planning**

		**Median**	**Range**
Volume at pl	Average	33.1	15.9/59.4
(cc)	H	32.6	16.0/58
	L	32.9	15.9/63.5
Mean D at pl	Weighted avr	35.7	17.5/57.4
(Gy)	H	39.9	27.4/70.4
	L	33.1	4.5/62.3
Shrinkage at mid tmt	Weighted avr	19.6	1.2/52.1
(%)	H	21.2	1.8/52.7
	L	19.0	0.7/51.6

*Abbreviations*: *pl* planning, *avr* average, *D* dose, *H/L* side that receives the higher (H) or lower (L) mean dose at planning within a given patient.

Median follow up is 35.8 months (range: 2.4-62.6 months). Six patients failed locoregionally and 5 more distantly. Locoregional control at 3 yrs is 90.4 ± 3.9%.

The median number of observations per patient is 4 (range: 1–7). All patients developed xerostomia: 8 (9.4%), 61 (71.8%) and 16 (18.8%) patients developed peak GR1, GR2 and GR3 dry mouth at some point, respectively. However, xerostomia improved in most patients with only 23 patients (27.0%) still considered having GR2+ dry mouth at the date of last follow up. From an endpoint perspective (GR2+), only 3 patients (3.5%) had inconsistent findings at consecutive follow ups (i.e. they were scored as having xerostomia resolution at an earlier follow up but subsequently relapsed to GR2+ toxicity at a later visit).

**Figure 1:**
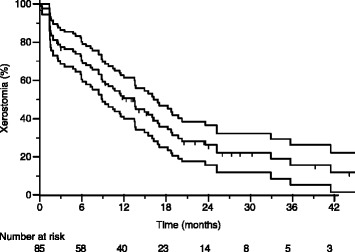
**Actuarial rate of physician-reported GR2+ xerostomia in the whole group of patients.**

The actuarial rate of GR2+ xerostomia is depicted in Figure 1 with rates of GR2+ xerostomia of 50.7% (SD: 5.5%), 26.2% (5.3%) and 15.9% (5.3%) at 1, 2 and 3 yrs, respectively.

Regarding the time to xerostomia resolution or downgrade to GR1, results of univariate analysis are reported in Table 3. Mid-treatment parotid shrink is weakly correlated to both BMI (rho = −0.234, p = 0.031) and pl-mean parotid D (rho = 0.258, p = 0.017). Results of multivariate analysis are reported in Table 4. Mid-treatment shrink along with weighted average mean parotid dose at planning and BMI are independent predictors of xerostomia resolution/downgrade to GR1.

**Table 3:** **Univariate analysis on the time to xerostomia resolution or downgrade to GR1**

**Covariate**	**Stratification**	**HR**	**95% CI**	**p value**
Age	Continuum	1.007	0.980-1.035	0.605
Sex	F vs M	0.679	0.243-1.900	0.461
AJCC stage	I-III vs IV	1.634	0.912-2.928	0.099
Induction + conc CHT	Yes vs No/Cet	0.519	0.162-1-659	0.269
Concomitant CHT	Yes vs No/Cet	0.556	0.295-1.050	0.070
Body Mass Index	Continuum	0.938	0.885-0.995	0.034
% PG Shrinkage at mid-tmt	Continuum	1.028	1.000-1.057	0.046
Smoking at diagnosis	Yes vs No	0.912	0.664-1.253	0.572
Smoking during follow up	Yes vs No	0.886	0.597-1.315	0.547
H- Mean PG pl-D	Continuum	0.962	0.930-0.995	0.023
L- Mean PG pl-D	Continuum	0.963	0.927-1.002	0.061
WA mean PG pl-D	Continuum	0.943	0.904-0.984	0.007
H- Mean SMG pl-D	Continuum	0.943	0.871-1.020	0.142
L- Mean SMG pl-D	Continuum	0.992	0.961-1.025	0.647
WA mean SMG pl-D	Continuum	0.981	0.935-1.030	0.436
WA mean PG + SMG pl-D	Continuum	0.944	0.904-0.987	0.011
Oral mucosa mean D	Continuum	0.991	0.951-1.033	0.674

*Abbreviations*: see Table 2; *HR* hazard ratio, *CI* confidence intervals, *CHT* chemotherapy, *pl-D* planning dose, *F* female, *M* male, *PG* parotid gland, *SMG* submandibular gland, *WA* weighted average.

**Table 4:** **Multivariate analysis**

**Covariate**	**HR**	**95%CI**		**p value**
		**Lower**	**Upper**	
Body Mass Index	0.932	0.875	0.992	0.027
% PG Shrinkage at mid-tmt	1.034	1.004	1.064	0.024
WA mean PG pl-D	0.927	0.886	0.971	0.001

Abbreviations: see Table 3.

**Figure 2:**
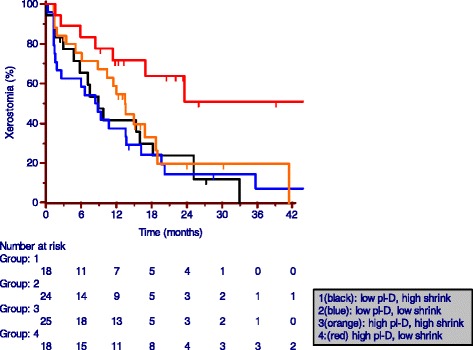
**Actuarial rate of physician-reported GR2+ xerostomia by both mid-treatment combined parotid gland shrinkage and mean weighted average parotid dose at planning.** Low/high represent values below/above median values of 19.6% and 35.7Gy for mid-treatment shrinkage and mean combo parotid dose, respectively.

Patients were pooled in 4 groups based on median values of both mid-treatment shrink (cut-off: 19.6%) and mean WA parotid pl-D (cut-off: 35.7 Gy). Figure 2 illustrates the time to xerostomia resolution/downgrade to GR1 by each group, disregarding BMI. Patients with a higher than median parotid dose at planning and who showed poor shrinkage at mid treatment are the ones with the worst outcome (3-yr rate of GR2+ xerostomia ≈ 50%); conversely, despite a higher than median parotid dose at planning, patients with an average mid treatment shrink larger than 19.6% had a time to xerostomia resolution/downgrade similar to that of patients planned to receive a lower mean dose to the parotids (for both subgroups, 3-yr rate of GR2+ xerostomia <10%). The difference between patients with high pl-D/low shrink and each of the other 3 groups is statistically significant (p < 0.05).

## Discussion

We have previously shown that mid-treatment parotid shrinkage is independently correlated to the planning mean dose [5]. The present paper shows that, once the mean dose at planning is accounted for, the percent shrinkage of parotids at mid-treatment predicts for the time to GR0-1 xerostomia. Therefore, as illustrated in Figure 2, mid treatment shrink helps to stratify patients with a higher than median parotid dose at planning into those at low and high risk of long-term GR2+ xerostomia.

This finding is somewhat unexpected and counterintuitive. Parotid shrinkage during treatment has been associated with a larger than planned delivered dose to the parotids due to their migration medially towards the high dose region [6]. Therefore, one would expect that the more the parotids shrink during treatment, the higher is the probability of long term side effects due to their overdosing compared to planning. On the other hand, in one study on 10 patients which assessed the cumulative dose received by the parotids, a higher than planned dose was reported only for a minority of patients [7]. Moreover, recent data shows that, on average, the extra mean dose delivered is modest (<4 Gy, overall) and, most importantly, other factors such as set-up errors are likely to have a higher impact on the final dose than parotid shrinkage [10].

In a previous study focusing on a small subset of the patients considered here (N = 24), we had found a positive correlation between the daily rate of both density and volume variations during the first two weeks of treatment and mean acute xerostomia scores according to CTCAE v3.0 [8]. Patients with rapidly shrinking parotids during the earlier part of treatment were those at higher risk of developing *acute* xerostomia. However, due to the limited number of patients, we could not tease out the effects of parotid shrinkage and mean parotid dose at planning which we had previously found to be directly correlated [5]. In the present study the higher number of patients included (N = 85) allowed us to analyze the effect of parotid shrinkage on long term xerostomia while ‘correcting’ for the dose at planning. At later time points and after accounting for the mean parotid dose, we found that the opposite is true: shrinkage during the first part of treatment predicts for a higher rate of long-term recovery.

Acinar cell loss is the main cause of functional damage in human salivary glands after RT and we previously found that parotid shrinkage during treatment is accompanied by a decrease in tissue density consistent with a relative increase in fat over glandular tissue [11,12]. Parotids that show modest volumetric changes earlier during treatment are at higher risk of persistent damage, suggesting that their acinar component is limited to begin with. Therefore, even if a given dose of radiation would kill the same fraction of cells, the absolute damage would be higher for those glands with a lower baseline acinar component. On the other hand, there is a clear possibility that more sensitive patients (i.e.: showing larger shrinkage) could experience a faster replacement of the acinar cells due to the activation of stem cells, efficiently recovering the gland functionality in the long run. In any case, our results are hypothesis generating and a validation prospective clinical study is underway. If confirmed, they open the possibility to have an extra tool (besides the planning dose) to identify patients at risk of persistent GR2+ xerostomia. It is also interesting to note that the present data also tend to reduce the clinical impact of adaptive strategies that aim at keeping the dose to the parotids stable during the course of IMRT, since patients who are supposed to benefit more from such strategies, those with significant parotid shrinkage, are the ones with the higher functional recovery after a non-adapted treatment course.

Few other aspects of the present paper deserve some comments. To our knowledge this is the first study that included in the analysis of xerostomia also BMI. The present data show that the time to recovery of xerostomia in patients who are overweight is longer than in patients who are not overweight and this may be related to the well know observation of a higher rate of dry mouth of patients with BMI > 30 Kg/m^2^ [13].

We acknowledge that xerostomia is not only due to parotid gland irradiation [14], though in the present study the dose to both the submandibular and minor salivary glands (oral mucosa dose) did not show independent predictive value on xerostomia suggesting that their role is probably marginal relatively to the endpoint considered here. Of note, contrary to Ortholan et al. [15], we found a higher predictive value when the mean parotid dose on both sides was combined over considering each side separately. Regarding the endpoint, it is well known that physician-reported outcomes do not strictly reflect patient-reported ones [16], the former being an interpretation of the latter ones. However, it should be noted that CTCAE criteria for xerostomia incorporate both subjective and objective assessments. According to Common Toxicity Criteria Adverse Event v 3.0+, what differentiates between grade 1 and 2 salivary gland toxicity is whether ‘significant oral intake alterations’ are present. These include ‘copious water’ and ‘other lubricants’ as well as dietary alterations (soft diet, moist foods) though the latter ones are usually related more to dysphagia than xerostomia.

## Conclusion

In conclusion, we provide evidence that, for a given radiation dose at planning, patients whose parotids significantly shrink during IMRT for oropharyngeal cancer are less likely to be dependent from supplemental fluids at 1 and 2 yrs after treatment. This finding potentially provides a novel tool to identify patients at risk in a timely way during treatment. Prospective validation is warranted and underway.
